# Material Extrusion Additive Manufacturing with Polyethylene Vitrimers

**DOI:** 10.3390/polym15061332

**Published:** 2023-03-07

**Authors:** Maria Camila Montoya-Ospina, Jiachen Zeng, Xiao Tan, Tim A. Osswald

**Affiliations:** Polymer Engineering Center, Department of Mechanical Engineering, University of Wisconsin-Madison, Madison, WI 53706, USA

**Keywords:** polyethylene, vitrimers, 3D printing, material extrusion, additive manufacturing, anisotropy, annealing

## Abstract

Polyethylene (PE) is one of the most widely used polymers in conventional polymer manufacturing processes. However, it remains a challenge to use PE in extrusion-based additive manufacturing (AM). Some of the challenges that this material presents include low self-adhesion and shrinkage during the printing process. These two issues lead to higher mechanical anisotropy when compared to other materials, along with poor dimensional accuracy and warpage. Vitrimers are a new class of polymers that have a dynamic crosslinked network, allowing the material to be healed and reprocessed. Prior studies on polyolefin vitrimers suggest that the crosslinks reduce the degree of crystallinity and increase the dimensional stability at elevated temperatures. In this study, high-density polyethylene (HDPE) and HDPE vitrimers (HDPE-V) were successfully processed using a screw-assisted 3D printer. It was demonstrated that HDPE-V were able to reduce shrinkage during the printing process. This shows that 3D printing with HDPE-V will provide better dimensional stability when compared to regular HDPE. Furthermore, after an annealing process, 3D-printed HDPE-V samples showed a decrease in mechanical anisotropy. This annealing process was only possible in HDPE-V due to their superior dimensional stability at elevated temperatures, with minimal deformation above melting temperature.

## 1. Introduction

Additive manufacturing (AM) is an advanced manufacturing technique that allows the fabrication of customized 3D objects with high geometric complexity that cannot be achieved with other processing techniques. The process consists of building the part in a layer-by-layer manner [1]. For several decades, AM has mainly been used for aesthetic and functional prototyping due to its cost-effectiveness and rapid prototyping. However, as innovative materials and AM methods are being developed, new applications are emerging in the field [2,3,4]. In general, these applications are shifting from prototypes to functional products [5,6]. Material extrusion (ME) is the most widely used AM technique, due to its low cost of fabrication and the availability of low-cost printers [3]. Material extrusion additive manufacturing (ME-AM) uses a relatively small number of working parts in the printing hardware, making it more user-friendly, and generally uses thermoplastics, which can reduce cost and allow for more freedom in material selection [4]. In ME-AM, the polymer is heated above the melting temperature for semicrystalline materials (and above the glass transition temperature for amorphous materials) and is dispensed through a nozzle. Once the polymer exits the nozzle, the viscosity sharply increases as it cools down to form a permanently bonded structure and retain the desired shape [3,7].

One significant limitation encountered by structures fabricated via ME-AM for functional applications is the decreased mechanical properties caused by anisotropy [8,9]. This is due to the weak bonds formed between layers during the printing process. Several studies have focused on improving the mechanical properties of printed parts via ME. Efforts have been made to understand the weld formation in ME-AM from the perspective of polymer interdiffusion [10]. Other works focusing on reducing the anisotropic properties of the 3D-printed parts include infrared preheating in polyphenylene sulfide parts [11], crosslink formation between layers in polylactic acid (PLA) parts [12], implementing thermoplastic supramolecular interactions in polyethylene terephthalate/phenylacetylene [13], and introducing Diels–Alder reactions based on furan–maleimide [14]. Low dimensional accuracy is another challenge in ME-AM and is related to warping, shrinkage, and delamination during the printing process [15,16,17,18,19]. These issues are exacerbated when 3D printing with semicrystalline materials. Common approaches to improve dimensional accuracy in ME-AM include increasing bed adhesion, reducing the degree of crystallinity, and optimizing the processing parameters [20].

Introducing novel materials in the ME-AM process, such as vitrimers, is a promising approach to tackle these challenges [21]. Vitrimers are a new class of covalent adaptable network (CAN) materials introduced by Leibler and co-workers in 2011 [22]. They consist of chemically crosslinked networks that engage in thermoactivated associative exchange reactions. During the exchange reactions, the network can change its topology while maintaining a constant degree of crosslinking [23]. Due to this constant degree of crosslinking, the structural integrity of the part is minimally affected when heat is applied. Furthermore, the dynamic crosslinking can provide shape memory, malleability, adhesion, and healing, unlike permanently crosslinked networks such as thermosets [23,24,25].

Vitrimers prepared from commercial thermoplastics can be utilized in ME-AM [26]. An excellent candidate thermoplastic to be transformed into a vitrimer is polyethylene (PE). PE is commonly used in a wide range of industrial and consumer applications due to its affordability, ease of processability, and high chemical resistance [27]. Nevertheless, PE has exhibited significant challenges in ME-AM processes; therefore, filaments for 3D printing are not widely available [19]. Due to its semicrystalline nature, PE tends to shrink during the filament manufacturing process, leading to low diametric consistency [28]. Additionally, PE has low adhesion to traditional metal and glass beds and tends to warp [29].

This study explores the printability of high-density polyethylene vitrimer (HDPE-V) using ME-AM as an approach to reduce mechanical anisotropy and improve dimensional accuracy. HDPE and HDPE-V pellets were used as the feedstock instead of a filament, due to the challenges that filament production of HDPE presents [28,30]. The crosslinking reaction that produced the HDPE-V presented in this study was obtained from the reaction between maleic -anhydride-grafted high-density polyethylene (HDPE-MAH) and a diamine crosslinker—4,4′-dithiodianiline (DTA). The concentration of MAH was 0.3% wt., and a molar ratio of 1:0.5 (MAH:DTA) was used. The reaction was conducted via a single-step process in a screw-assisted 3D printer at 220 °C. Pellet-fed screw-assisted 3D printers are increasingly being used to bypass the need for filaments, reducing the associated cost of filament production, while also increasing the deposition rate and expanding the range of 3D-printing materials [31]. For example, modified 3D printers have enabled the use of recycled polymer flakes from polyethylene terephthalate (PET) water bottles [32], polymer composites that are too brittle to be spooled into filaments [33], and recycled selective laser sintering (SLS) powder [34].

In a previous study, it was shown that vitrimers prepared from HDPE (HDPE-V) significantly affect the properties of the material in the melt and solid states [35]. In the melt state, the crosslinks in HDPE-V were responsible for its superior dimensional stability at elevated temperatures, due to the presence of a rubbery plateau compared to un-crosslinked HDPE. In the solid state, it was observed that the crosslinks hindered the crystallization of the material. The objective of this study was to apply these findings to demonstrate that HDPE-V is a promising material for ME-AM. This research shows that shrinkage and mechanical anisotropy were decreased when using HDPE-V in an ME-AM process compared to HDPE. The reduced degree of crystallinity in HDPE-V played a role in the reduced shrinkage of the parts. An improvement in mechanical anisotropy was observed in HDPE-V, and this was achieved via a thermal post-processing step. The annealing step was only possible because of the enhanced dimensional stability of HDPE-V at elevated temperatures.

## 2. Materials and Methods

### 2.1. Materials

High-density polyethylene (HDPE) F04660 (MFI = 0.7 g/10 min at 190 °C with 2.16 kg) and maleic-anhydride-grafted high-density polyethylene (HDPE-MAH) were supplied by SABIC (Geleen, The Netherlands). The grafting process of MAH is proprietary information. The MAH concentration used in this study was 0.3% wt. The crosslinker used to produce the HDPE vitrimer (HDPE-V)—4,4′-dithiodianiline (DTA)—was purchased from Tokyo Chemical Industry (Tokyo, Japan). A summary of the relevant material properties provided by the suppliers is given in Table 1.

**Figure 1:**
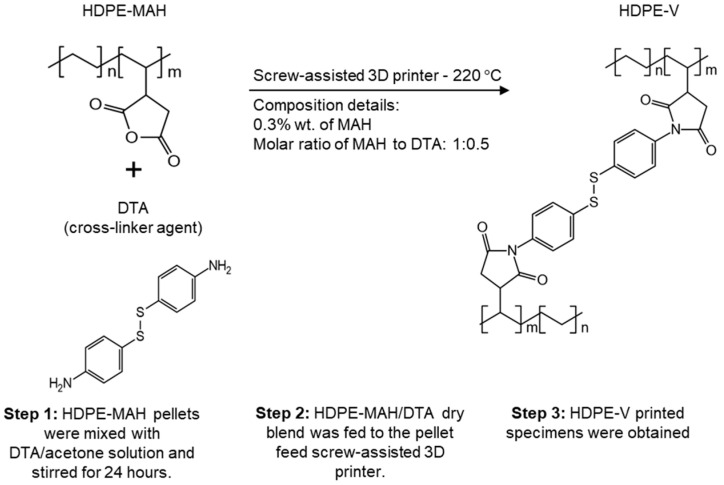
Graphical representation of the protocol used to produce HDPE-V using ME-AM.

HDPE-V was obtained from the reaction of HDPE-MAH and the diamine crosslinker containing disulfide bonds—4,4′ dithiodianiline (DTA)—as described in a previous work [35]. The concentration of MAH was 0.3% wt., and a molar ratio of 1:0.5 (MAH:DTA) was used. The HDPE-V feedstock for the 3D printer was prepared by dissolving the crosslinker powder (DTA) in acetone, and HDPE-MAH pellets were coated in this solution at room temperature. The solution was constantly stirred for 24 h to ensure evenly coated pellets. After this period, the acetone evaporated, and the pellets were fully dried. This dry blend was then introduced to the (pellet-fed) screw-assisted 3D printer, where the reaction took place in the melt state to form HDPE-V. This procedure is summarized in Figure 1.

### 2.2. Material Extrusion Additive Manufacturing and Geometric Design

**Figure 2:**
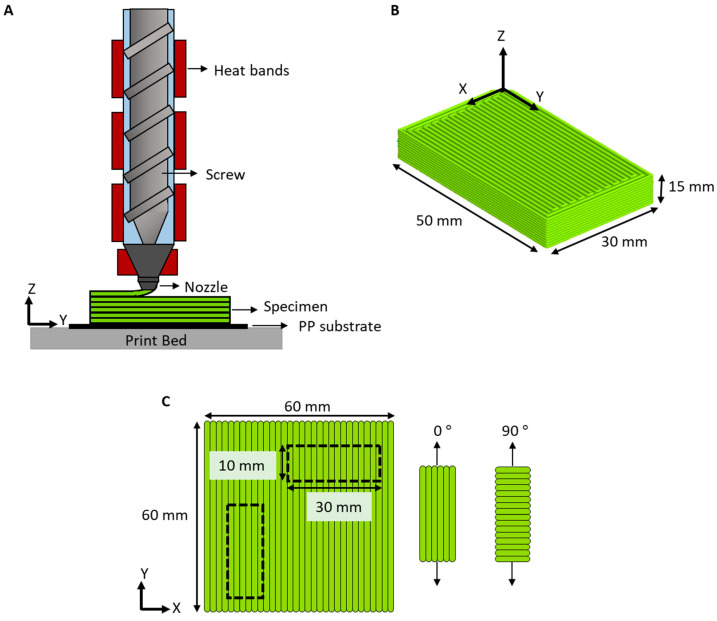
(**A**) Schematic of screw-assisted ME-AM; (**B**) sample geometry used for microstructure characterization; (**C**) specimens used for viscoelastic characterization (H: 3 mm).

A screw-assisted 3D printer was used to produce all test specimens. The machine used in this study was a Cosine AM1 (Cosine Additive, Houston, TX, USA) with a pellet-fed extruder attachment. This configuration allows the extrusion of materials without the need to manufacture filaments as the feedstock. The pellets were fed through a hopper and then transported and melted through the heated single screw. The material was pushed through the nozzle and deposited layer-by-layer on a polypropylene (PP) substrate, as depicted in Figure 2A. PP was used as a substrate, as it has been proven to improve bed adhesion and, therefore, reduce delamination [30].

Rectangular specimens used for examining the microstructure were produced with dimensions of H: 15 mm × W: 30 mm × L: 50 mm, as shown in Figure 2B. Square plates (H: 3 mm × W: 60 mm × L: 60 mm) were also produced to evaluate shrinkage, thermal, rheological, and viscoelastic properties. Smaller rectangular specimens (10 mm × 30 mm) were punched from the square plates, as depicted in Figure 2C, to evaluate the viscoelastic behavior of the parts with varying bead orientation.

The specimens shown in Figure 2B,C were generated through computer-aided design using SOLIDWORKS 2021 and were translated into G-Code using Simplify 3D software (Version 4.1.2).

### 2.3. Shrinkage Evaluation

Shrinkage perpendicular to bead orientation and parallel to bead orientation was measured by comparing the original dimensions in the X-Y plane of the specimens shown in Figure 2C (60 mm × 60 mm) to the final dimensions of the printed specimens. The final dimensions were measured after 48 h of printing. Equation (1) refers to the shrinkage perpendicular to bead orientation ($S_{w}$), while Equation (2) refers to the shrinkage parallel to bead orientation ($S_{l}$) [36].

(1) $$S_{w}=\frac{W_{o}-W_{m}}{W_{o}}\times 100$$

(2) $$S_{l}=\frac{L_{o}-L_{m}}{L_{o}}\times 100$$

where

$W_{o}$ = original dimensions (from CAD) perpendicular to bead orientation;

$W_{m}$ = measured dimensions perpendicular to bead orientation;

$L_{o}$ = original dimensions (from CAD) parallel to bead orientation;

$L_{m}$ = measured dimensions parallel to bead orientation.

### 2.4. Characterization

Differential scanning calorimetry (DSC) experiments were performed with a 214 Polyma DSC (NETZSCH, Selb, Germany). Two heating cycles were conducted in the range of 30–200 °C at a heating rate of 10 °C/min in a nitrogen atmosphere. The melting and crystallization properties were determined from the second heating and cooling cycle. The degree of crystallinity (χ_c_) was calculated from the ratio of the measured melting enthalpy (ΔH_m_) and the theoretical melting enthalpy of 100% crystalline polyethylene (293 J/g) [37].

Rheological tests were conducted using an AR 2000 ex rheometer (TA Instruments, New Castle, DE, USA). A 25 mm parallel steel plate fixture was used with a 1.85 mm gap. Frequency sweeps were performed over a range of 0.01 to 100 rad/s at 220 °C and 0.1% strain.

Dynamic mechanical analysis (DMA) was conducted in an Explexor 500 N DMA (NETZSCH, Selb, Germany) to measure the viscoelastic properties of the 3D-printed specimens. Frequency sweeps were performed at room temperature in tension within the linear viscoelastic regime, using a dynamic strain of 0.03% and a frequency range of 0.5–100 Hz.

Scanning electron microscopy (SEM) was used to identify voids in the 3D-printed specimens. The 3D-printed samples were examined using a Hitachi S3400 Variable-Pressure Scanning Electron Microscope at 15 kV and 30 Pa (Hitachi, Tokyo, Japan).

### 2.5. Annealing Procedure

A post-processing treatment was conducted with the purpose of reducing mechanical anisotropy by improving chain diffusion between layers. The 3D-printed specimens used in DMA testing were heat-treated for 10 min at 150 °C in an oven under normal atmospheric conditions. A prototype was printed and annealed to visually demonstrate the dimensional stability of the parts during the heat treatment process. 

## 3. Results and Discussion

### 3.1. Assessment of Processability in Screw-Assisted AM

**Figure 3:**
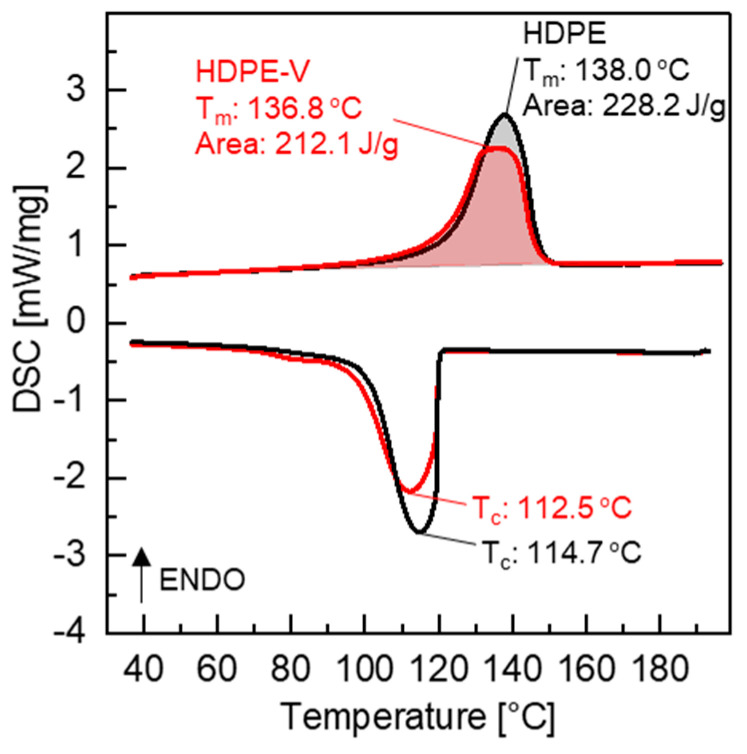
DSC heating and cooling curves (10 °C/min in nitrogen).

Differential scanning calorimetry was utilized to determine the melting temperature, crystallization temperature, and heat of fusion of the materials to be printed. The results from the second heating and cooling cycle are shown in Figure 3. The melting temperature of HDPE and HDPE-V was 138.0 °C and 136.8 °C, respectively. The crystallization temperature of HDPE and HDPE-V was 114.7 °C and 112.5 °C, respectively. Since the difference in both transition temperatures was small (<2.2 °C), the temperature profile of the printing process was chosen to be the same for both materials, as summarized in Table 2. The heat of fusion of HDPE-V was decreased by 16.1 J/g compared to HDPE. The heat of fusion was used to determine the degree of crystallinity of the samples: 77.9% (HDPE) and 72.4% (HDPE-V). Lower crystallinity can be beneficial for 3D-printing processes, as it can reduce the shrinkage of samples and improve their dimensional accuracy [18,29].

**Figure 4:**
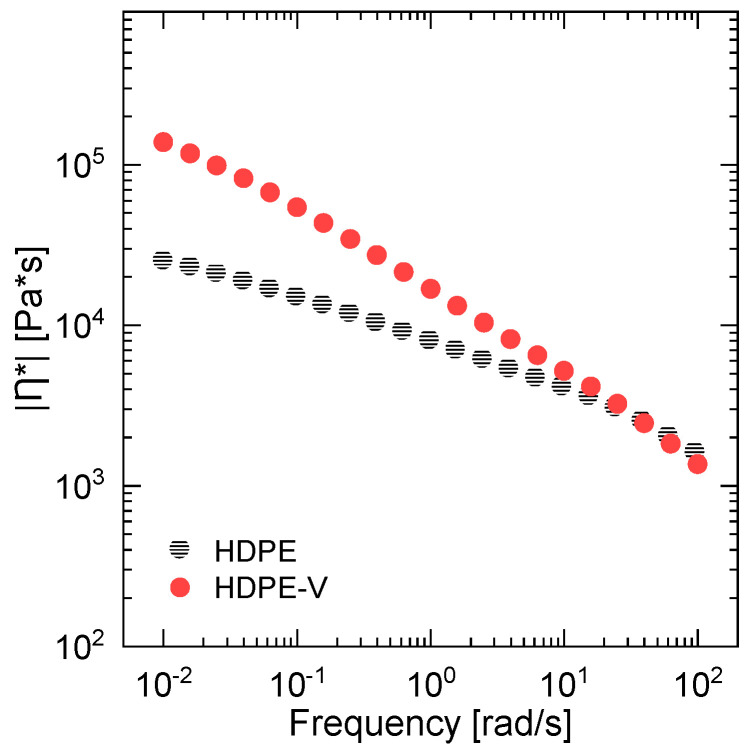
Complex viscosity $\left|\eta^{*}\right|$ as a function of the frequency of HDPE and HDPE-V (220 °C).

The rheological behavior of HPDE and HDPE-V was studied to assess the processability of the materials and tune the parameters in the screw-assisted 3D printer. The measurements were conducted in a parallel-plate rheometer within the linear viscoelastic regime at 220 °C. This temperature was chosen because it is well above the melting temperature of both materials and falls within the range of typical temperatures used for 3D printing of PE [19]. HDPE-V shows higher complex viscosity $\left|\eta^{*}\right|$ at low frequency (0.01–10 rad/s) compared to HDPE (Figure 4). The high viscosity seen at the lower end of the frequency range tested was due to the presence of the characteristic crosslinked network of the vitrimer. However, in the range of 10–100 rad/s, the complex viscosity values of HDPE and HDPE-V are very comparable. Since the typical shear rate experienced in screw-assisted 3D printing is usually higher than 10 s^−1^, it can be concluded that both materials in this study can be processed at the same temperature [7,14].

Figure 4 also reveals the shear-thinning behavior of both polymers. The extent of shear thinning was estimated by fitting the data in Figure 4 to the power-law model (Equation (3)), where K is the consistency index and n is the power-law index. Low values of n indicate a stronger shear-thinning behavior. The calculated values of n of HDPE and HDPE-V were 0.59 and 0.41, respectively. Therefore, the vitrimer has a higher shear-thinning dependency, which is usually desirable in extrusion-based 3D-printing processes. Higher shear thinning is usually desired for two reasons: (1) the polymer extruded through the nozzle (high shear rate) should have high flowability, which translates into low viscosity values; and (2) during the deposition step (low shear rate), the polymer should have high viscosity to hold its shape under gravity and under the layers on top [7,38].

(3) $$\eta =K\times {\dot{\gamma }}^{n-1}$$

### 3.2. Printing Challenges

Adhesion was the first challenge encountered when printing with HDPE and HDPE-V. At room temperature, neither of the materials adhered to the aluminum bed substrate of the AM1 Cosine. Previous studies have shown that adhesion between PE parts and the bed can be improved by (1) increasing the build temperature to prevent solidification of the first layer, or (2) by selecting an appropriate build material [17]. Some examples of build materials include polypropylene (PP) [30], ultra-high molecular weight polyethylene (UHMWPE) [39], or styrene-ethylene-butylene-styrene (SEBS) sheets [29]. As a first attempt to improve bed adhesion, the build temperature was set to 125 °C. This would ensure that the material was above the crystallization temperature, which could mitigate the shrinkage and warpage that takes place when the material cools down. Additionally, this would delay the crystallization, which could improve the polymer chain diffusion between the beads, leading to lower mechanical anisotropy in the printed parts [40]. It was decided to implement this method first due to the potential benefits this could bring to the final parts’ properties. However, a high print bed temperature created a melt pool in the first two layers of the printed material due to its low viscosity. Since print quality was an issue, another approach to improve bed adhesion was utilized. The temperature of the bed was decreased to 60 °C, and a PP substrate was used. A 1/16 inch PP sheet and a Magigoo PP adhesive led to a significant increase in bed adhesion.

**Figure 5:**
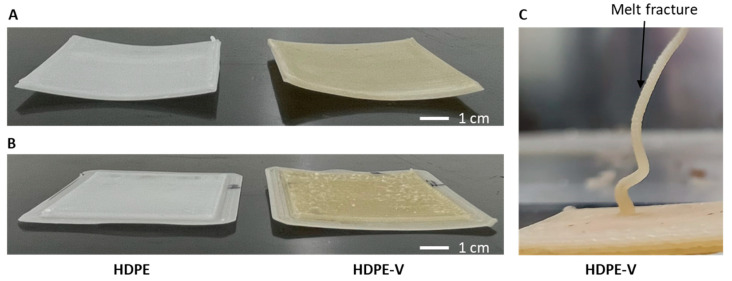
Square plates (60 mm × 60 mm × 3 mm) produced (**A**) without brims and (**B**) with brims. (**C**) Melt fracture observed in HDPE-V.

Additionally, a brim was added to the parts to further increase their adhesion. Incorporating a brim around the part increases the interface of the print object with the substrate. This can lower the debonding stresses and, consequently, decrease warpage. Previous studies successfully reduced warpage when using a brim with five lines [19,41]. Additionally, Bachhar et al. showed that a 5–15-line brim did not change the warpage significantly [41]. A five-line brim was sufficient to prevent delamination of the specimens presented in this study. A comparison of the printed parts without brims is shown in Figure 5A, while those with brims are shown in Figure 5B.

**Figure 6:**
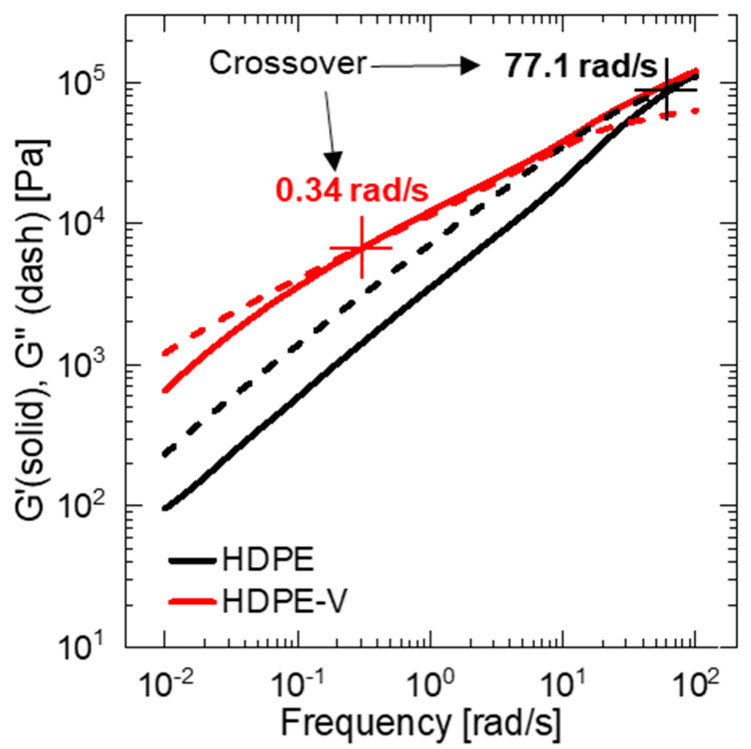
Dynamic moduli as a function of frequency for HDPE (black lines) and HDPE-V (red lines) at 220 °C. Storage modulus (G′) is represented by the solid lines and viscous modulus (G″) is represented by the dashed lines.

The second challenge observed when printing with HDPE-V was melt distortion, more commonly known as melt fracture. This can be seen as a rough surface finish in Figure 5C. Melt fracture is a type of flow instability that is common in extrusion operations. At low shear rates, a stable, smooth stream at the exit of the die is usually observed in polymer extrusion operations. However, at higher shear rates, the extrudate can become distorted, and this depends on the type of polymer being extruded. Furthermore, there is an agreement that melt elasticity, which is measurable by the storage modulus (G′), plays a major role in the initiation of this type of flow instability [42,43]. The higher G′ values of HDPE-V compared to HDPE explain why melt fracture is initiated in the former material (solid lines in Figure 6). The melt elasticity in HDPE-V is a consequence of the crosslinked network.

**Figure 7:**
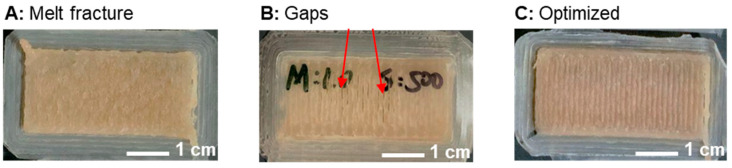
Print speed and multiplier optimization for HDPE-V to reduce melt fracture. (**A**) Speed: 800 mm/min; multiplier: 1×. (**B**) Speed: 500 mm/min; multiplier: 1×. (**C**) Speed: 500 mm/min; multiplier: 1.2×.

It was important to reduce or eliminate melt fracture to avoid introducing voids between the layers. Melt fracture, usually shown as surface distortions, occurs when the polymer melt exits the die at throughput rates above a critical value. Therefore, the extrusion speed was reduced from 800 mm/min to 500 mm/min, and the multiplier was set to 1×. By decreasing the extrusion speed, the screw rotational speed (rpm)—and, therefore, the throughput rate—was also decreased. Demonstration parts are shown in Figure 7A,B, where an improvement in the surface finish can be seen. However, reducing the throughput led to under–extrusion, as can be observed from the gaps shown in Figure 7B with the red arrows. The multiplier was increased to 1.2×, which resolved this issue (Figure 7C). It is important to note that reducing the print speed could lead to two advantages relevant to this work: (1) it can reduce melt fracture, and (2) it can increase the weld time between beads, which can promote interlayer adhesion [10].

Since ME-AM printing parameters can affect the final parts’ properties, it was essential that all 3D-printed specimens were manufactured under the same printing conditions. As previously discussed, the only parameter that required modification was the print speed. When a print speed of 800 mm/min was used, HDPE did not show any flow distortion, and the surface of the bead was smooth. However, HDPE-V exhibited flow distortions at this print speed. HDPE-V is susceptible to melt fracture due to its crosslinked network and melt elasticity [42,43]. For this reason, the print speed was decreased until no distortion was observed. The final print speed used for all of the specimens was 500 mm/min. Finally, the optimized printing parameters used for both materials (HDPE and HDPE-V) are summarized in Table 2.

### 3.3. Shrinkage

During cooling, polymers experience a decrease in free volume between their macromolecular chains, which leads to shrinkage. The extent of shrinkage is greater in semicrystalline polymers such as PE due to their ability to crystallize [44]. Shrinkage can have a significant impact on the dimensional accuracy and the appearance of the final product. Any 3D-printed parts that shrink in an anisotropic manner could lead to potential issues during and after the printing process. Parts with different amounts of shrinkage in the flow and transverse flow directions can lead to part distortion. The undesirable deformation, usually referred to as warpage, is caused by residual stresses that are created during cooling [20]. Furthermore, warpage can lead to delamination during printing and, therefore, to print failures. Even if delamination could be avoided during the printing step, the dimensional accuracy of the final part would be affected. This could lead to issues during assembly or end-use application.

Shrinkage perpendicular $\left(S_{w}\right)$ and parallel $\left(S_{l}\right)$ to the print orientation was determined in HDPE and HDPE-V. From Table 3, it can be observed that HDPE-V experienced less shrinkage in both directions in comparison to HDPE. This result can be explained by the lower degree of crystallinity of HDPE-V (72.4%) compared to HDPE (77.9%). Additionally, both materials shrink in an anisotropic manner ($S_{l}$ > $S_{w}$). This behavior could be a result of the induced molecular orientation upon shear flow during extrusion [45].

### 3.4. Mechanical Properties

**Figure 8:**
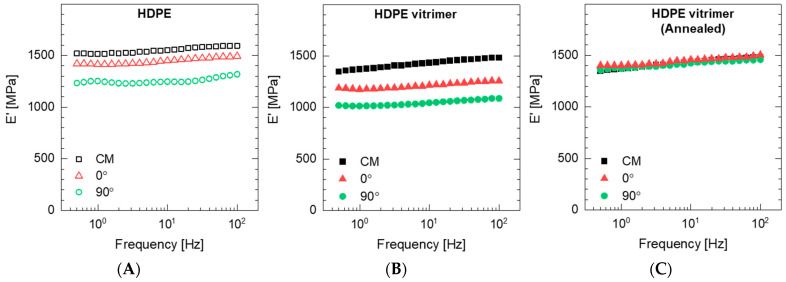
DMA frequency sweeps of compression-molded (CM) samples and 3D-printed specimens with loading parallel to the bead orientation (0°) and perpendicular to the bead orientation (90°): (**A**) HDPE, (**B**) HDPE-V, and (**C**) HDPE-V after an annealing process.

The viscoelastic behavior of untreated and annealed samples was determined using DMA under tension loading. Measurements were conducted at room temperature with the loading parallel (0°) and perpendicular (90°) to the print direction. Samples manufactured via compression molding were included for comparison purposes. Figure 8A,B show the storage modulus (E′) of HPDE and HDPE-V specimens, respectively. For all samples, E′ increased with increasing frequency. This is consistent with the time-dependent behavior of polymers in response to deformation. At higher frequencies or smaller timescales, these materials behave more like solids, as characterized by higher E′, whereas at lower frequencies or smaller timescales the samples behave more like fluids, as shown by lower E′.

Specimens manufactured via compression molding exhibited the highest modulus, followed by 3D-printed samples in the 0° and 90° orientations. For all samples, HDPE exhibited higher E′ compared to HDPE-V. Previous research has demonstrated a linear relationship between crystallinity and stiffness [35]. Higher crystallinity in HDPE explains its higher modulus compared to HDPE-V. HDPE samples tested in the 0° orientation showed a drop of approximately 100 MPa in E′ relative to compression-molded HDPE, while the 90° orientation showed a drop of around 300 MPa. HDPE-V printed with 0° and 90° orientations showed a drop of approximately 250 MPa and 350 MPa in E′, respectively, relative to the compression-molded sample in the entire frequency range.

Both materials displayed mechanical anisotropy consistent with material extrusion 3D printing. However, HDPE showed a lower degree of anisotropy compared to HDPE-V. This can be explained by the stronger interlayer adhesion in HDPE samples. Improved interlayer adhesion is expected if the chain interdiffusion is promoted [10]. The latter can happen if the viscous modulus (G″) of the melt dominates over the elastic modulus (G′) in the terminal region. Both materials satisfied the condition of G″ > G′ in the terminal region (Figure 6). However, the ratio of G″ to G′ of HDPE and HDPE-V in the terminal region was 2.5 and 1.9, respectively. Hence, a higher interlayer adhesion is expected in HDPE.

**Figure 9:**
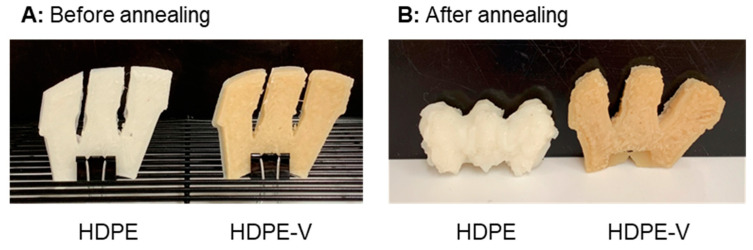
Visual demonstration of the annealing process: (**A**) before placing the parts in the oven, and (**B**) after 10 min in the oven at 150 °C.

A thermal post-processing treatment was conducted to improve the chain interdiffusion between layers and reduce the mechanical anisotropy. In the context of Figure 8, lower mechanical anisotropy refers to narrowing the gap between the storage modulus (G′) in the 0° and 90° orientations. A temperature closest to the end of melting of HDPE and HDPE-V was chosen to ensure the destruction of crystallites and to enhance chain mobility. As previously observed from the DSC scans (Figure 3), both HDPE and HDPE-V samples were fully melted at 150 °C. When observing the samples being heated in the oven, it was revealed that the entire sample melted after 10 min. After annealing the HDPE specimens used in the DMA testing, the samples were completely deformed, while the HDPE-V specimens suffered comparatively minor changes in dimensions. A visual demonstration of the annealing process conducted in a prototype part is shown in Figure 9. Although in the present study the mechanical anisotropy was dramatically reduced in HDPE-V—as observed by the increase in modulus in the 0° and 90° orientations (Figure 8C)—future studies should focus on improving the annealing methodology. An improved post-annealing dimensional accuracy will enable the usage of HDPE-V in ME-AM processes to manufacture functional isotropic parts.

### 3.5. Microstructures

**Figure 10:**
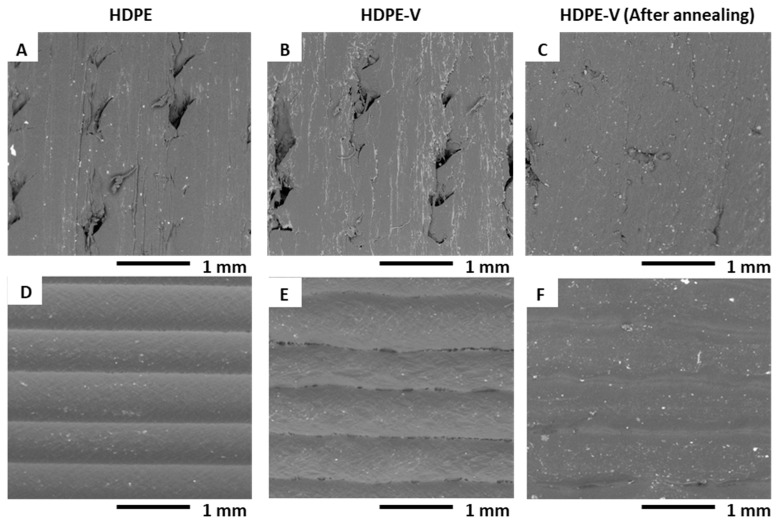
SEM imaging of the samples shown in Figure 2B: (**A**–**C**) 3D-printed samples normal to the print direction (X-Z view in Figure 2B), and (**D**–**F**) 3D-printed samples parallel to the print direction (Y-Z view in Figure 2B).

The microstructure of the samples was observed using SEM to elucidate their interlayer adhesion and the presence of voids. Figure 10A–C depict the SEM micrographs of the 3D-printed samples normal to the print direction (X-Z view of the samples shown in Figure 2B). Similarly, Figure 10D–F show the SEM micrographs of the 3D-printed samples parallel to the print direction (Y-Z view of the samples shown in Figure 2B). Interbead gaps characteristic of ME-AM were observed in the cross-section of HDPE and HDPE-V. Furthermore, small voids were observed in the HDPE-V samples parallel to the print direction (Figure 10E). These voids could have been introduced during the deposition step. This could be a result of the high viscosity of HDPE-V, which hindered the formation of a strong weld. The presence of these small voids could also contribute to the mechanical anisotropy found in HDPE-V (Figure 8B). Finally, after annealing, the interbead gaps were dramatically reduced and the layer adhesion was improved, as shown in Figure 10C.

## 4. Conclusions

Material extrusion 3D printing of HDPE and HDPE vitrimers was conducted. The main achievements of this work are as follows:

- Bed adhesion of HDPE and HDPE vitrimer parts was improved by using a PP bed substrate.
- Extrudate distortion (melt fracture) in HDPE vitrimers was resolved by decreasing the print speed.
- Rheological measurements indicated that the vitrimer has a higher shear-thinning dependency, which is usually desired in extrusion-based 3D-printing process.
- Thermal measurements demonstrated that HDPE vitrimers had a lower degree of crystallinity, which led to lower shrinkage during printing and increased dimensional accuracy.
- Viscoelastic measurements revealed the mechanical anisotropy of parts consistent with material extrusion 3D-printing processes. Interlayer adhesion was improved, and the void content was reduced in HDPE-V after a thermal post-processing step. In consequence, mechanical anisotropy was significantly reduced in HDPE-V.

## Figures and Tables

**Table 1:** Characteristic properties of the materials used in this study.

Material	Melting Temperature (°C)	Density (g/cm^3^)	Molecular Weight (g/mol)
HDPE F04660	134	0.961	-
DTA	77	-	248.37

**Table 2:** Printing parameters of HDPE and HDPE-V.

Parameters	Value
Nozzle diameter	1 mm
Extruder temperature	220 °C
Nozzle temperature	220 °C
Bed temperature	60 °C
Printing speed	500 mm/min
Extrusion multiplier	1.2
Layer height	0.6 mm
Infill percentage	100%
First layer setting	Height 50%; speed 60%
Brim	5 layers
Substrate	PP sheet

**Table 3:** Shrinkage perpendicular ($S_{w}$) and parallel ($S_{l}$) to the print orientation in the plates shown in Figure 2C.

Sample	$S_{w}\;(\%)$	$S_{l}\;(\%)$
HDPE	0.91 ± 0.13	2.62 ± 0.15
HDPE-V	0.08 ± 0.03	1.87 ± 0.12

## Data Availability

Not applicable.

## References

[1] (2015). Standard Terminology for Additive Manufacturing—General Principles—Terminology.

[2] Love L., Post B., Noakes M., Nycz A., Kunc V. (2021). There’s Plenty of Room at the Top. Addit. Manuf..

[3] Jiang Z., Diggle B., Tan M.L., Viktorova J., Bennett C.W., Connal L.A. (2020). Extrusion 3D Printing of Polymeric Materials with Advanced Properties. Adv. Sci..

[4] Black H.T., Celina M.C., Mcelhanon J.R. (2016). Additive Manufacturing of Polymers: Materials Opportunities and Emerging Applications.

[5] Sanchez-Rexach E., Johnston T.G., Jehanno C., Sardon H., Nelson A. (2020). Sustainable Materials and Chemical Processes for Additive Manufacturing. Chem. Mater..

[6] Ngo T.D., Kashani A., Imbalzano G., Nguyen K.T.Q., Hui D. (2018). Additive Manufacturing (3D Printing): A Review of Materials, Methods, Applications and Challenges. Compos. Part B Eng..

[7] Arrigo R., Frache A. (2022). FDM Printability of PLA Based-Materials: The Key Role of the Rheological Behavior. Polymers.

[8] Gao X., Qi S., Kuang X., Su Y., Li J., Wang D. (2021). Fused Filament Fabrication of Polymer Materials: A Review of Interlayer Bond. Addit. Manuf..

[9] Colón Quintana J.L., Redmann A., Mazzei Capote G.A., Pérez-Irizarry A., Bechara A., Osswald T.A., Lakes R. (2019). Viscoelastic Properties of Fused Filament Fabrication Parts. Addit. Manuf..

[10] Seppala J.E., Hoon Han S., Hillgartner K.E., Davis C.S., Migler K.B. (2017). Weld Formation during Material Extrusion Additive Manufacturing. Soft Matter.

[11] Kishore V., Ajinjeru C., Nycz A., Post B., Lindahl J., Kunc V., Duty C. (2017). Infrared Preheating to Improve Interlayer Strength of Big Area Additive Manufacturing (BAAM) Components. Addit. Manuf..

[12] Levenhagen N.P., Dadmun M.D. (2019). Reactive Processing in Extrusion-Based 3D Printing to Improve Isotropy and Mechanical Properties. Macromolecules.

[13] Chen L., Zhao H.B., Ni Y.P., Fu T., Wu W.S., Wang X.L., Wang Y.Z. (2019). 3D Printable Robust Shape Memory PET Copolyesters with Fire Safety: Via π-Stacking and Synergistic Crosslinking. J. Mater. Chem. A.

[14] Yang K., Grant J.C., Lamey P., Joshi-Imre A., Lund B.R., Smaldone R.A., Voit W. (2017). Diels–Alder Reversible Thermoset 3D Printing: Isotropic Thermoset Polymers via Fused Filament Fabrication. Adv. Funct. Mater..

[15] Abbott A., Gibson T., Tandon G.P., Hu L., Avakian R., Baur J., Koerner H. (2021). Melt Extrusion and Additive Manufacturing of a Thermosetting Polyimide. Addit. Manuf..

[16] Kim S., Baid H., Hassen A., Kumar A., Lindahl J., Hoskins D., Ajinjeru C., Duty C., Yeole P., Vaidya U. Analysis on Part Distortion and Residual Stress in Big Area Additive Manufacturing with Carbon Fiber-Reinforced Thermoplastic Using Dehomogenization Technique. Proceedings of the CAMX 2019—Composites and Advanced Materials Expo.

[17] Jin M., Giesa R., Neuber C., Schmidt H.W. (2018). Filament Materials Screening for FDM 3D Printing by Means of Injection-Molded Short Rods. Macromol. Mater. Eng..

[18] Koffi A., Toubal L., Jin M., Koffi D., Döpper F., Schmidt H., Neuber C. (2022). Extrusion-based 3D Printing with High-density Polyethylene Birch-fiber Composites. J. Appl. Polym. Sci..

[19] Gudadhe A., Bachhar N., Kumar A., Andrade P., Kumaraswamy G. (2019). Three-Dimensional Printing with Waste High-Density Polyethylene. ACS Appl. Polym. Mater..

[20] Spoerk M., Holzer C., Gonzalez-Gutierrez J. (2020). Material Extrusion-Based Additive Manufacturing of Polypropylene: A Review on How to Improve Dimensional Inaccuracy and Warpage. J. Appl. Polym. Sci..

[21] Zou W., Dong J., Luo Y., Zhao Q., Xie T. (2017). Dynamic Covalent Polymer Networks: From Old Chemistry to Modern Day Innovations. Adv. Mater..

[22] Montarnal D., Capelot M., Tournilhac F., Leibler L. (2011). Silica-like Malleable Materials from Permanent Organic Networks. Science.

[23] Röttger M., Domenech T., Van Der Weegen R., Breuillac A., Nicolaÿ R., Leibler L. (2017). High-Performance Vitrimers from Commodity Thermoplastics through Dioxaborolane Metathesis. Science.

[24] Denissen W., Rivero G., Nicolaÿ R., Leibler L., Winne J.M., Du Prez F.E. (2015). Vinylogous Urethane Vitrimers. Adv. Funct. Mater..

[25] Imbernon L., Norvez S., Leibler L. (2016). Stress Relaxation and Self-Adhesion of Rubbers with Exchangeable Links. Macromolecules.

[26] Zander N.E., Seppala J.E., Kotula A.P., Snyder C.R. (2019). Recycled Polymer Feedstock for Material Extrusion Additive Manufacturing. Polymer-Based Additive Manufacturing: Recent Developments.

[27] Peacock A.J. (2000). Handbook of Polyethylene: Structures, Properties, and Applications.

[28] Baechler C., Devuono M., Pearce J.M. (2013). Distributed Recycling of Waste Polymer into RepRap Feedstock. Rapid Prototyp. J..

[29] Schirmeister C.G., Hees T., Licht E.H., Mülhaupt R. (2019). 3D Printing of High Density Polyethylene by Fused Filament Fabrication. Addit. Manuf..

[30] Chong S., Pan G.T., Khalid M., Yang T.C.K., Hung S.T., Huang C.M. (2017). Physical Characterization and Pre-Assessment of Recycled High-Density Polyethylene as 3D Printing Material. J. Polym. Environ..

[31] Justino Netto J.M., Idogava H.T., Frezzatto Santos L.E., de Castro Silveira Z., Romio P., Alves J.L. (2021). Screw-Assisted 3D Printing with Granulated Materials: A Systematic Review. Int. J. Adv. Manuf. Technol..

[32] Little H.A., Tanikella N.G., Reich M.J., Fiedler M.J., Snabes S.L., Pearce J.M. (2020). Towards Distributed Recycling with Additive Manufacturing of PET Flake Feedstocks. Materials.

[33] Capote G.A.M., Montoya-Ospina M.C., Liu Z., Mattei M.S., Liu B., Delgado A.P., Yu Z., Goldsmith R.H., Osswald T.A. (2022). Compounding a High-Permittivity Thermoplastic Material and Its Applicability in Manufacturing of Microwave Photonic Crystals. Materials.

[34] Silveira Z.D., de Freitas M.S., Inforçatti Neto P., Noritomi P.Y., da Silva J.V. (2014). Design Development and Functional Validation of an Interchangeable Head Based on Mini Screw Extrusion Applied in an Experimental Desktop 3-D Printer. Int. J. Rapid Manuf..

[35] Montoya-Ospina M.C., Verhoogt H., Ordner M., Tan X., Osswald T.A. (2022). Effect of Cross-Linking on the Mechanical Properties, Degree of Crystallinity and Thermal Stability of Polyethylene Vitrimers. Polym. Eng. Sci..

[36] (2021). Standard Test Method of Measuring Shrinkage from Mold Dimensions of Thermoplastics.

[37] Menczel J.D., Prime R.B. (2009). Thermal Analysis of Polymers: Fundamentals and Applications.

[38] Jain T., Tseng Y.M., Tantisuwanno C., Menefee J., Shahrokhian A., Isayeva I., Joy A. (2021). Synthesis, Rheology, and Assessment of 3D Printability of Multifunctional Polyesters for Extrusion-Based Direct-Write 3D Printing. ACS Appl. Polym. Mater..

[39] Spoerk M., Gonzalez-Gutierrez J., Lichal C., Cajner H., Berger G.R., Schuschnigg S., Cardon L., Holzer C. (2018). Optimisation of the Adhesion of Polypropylene-Based Materials during Extrusion-Based Additive Manufacturing. Polymers.

[40] Shang Y., Xu Q., Jiang B., Yang Y., Liu X., Jiang Z., Yu C., Li X., Zhang H. (2022). Slowing Crystallization to Enhance Interlayer Strength of 3D Printed Poly (Ether Ether Ketone) Parts by Molecular Design. Addit. Manuf..

[41] Bachhar N., Gudadhe A., Kumar A., Andrade P., Kumaraswamy G. (2020). 3D Printing of Semicrystalline Polypropylene: Towards Eliminating Warpage of Printed Objects. Bull. Mater. Sci..

[42] Blyler L., Hart A.C. (1970). Capillary Flow Instability of Ethylene Polymer Melts. Poly. Eng. Sci..

[43] Kim Y.C., Yang K.S. (1999). Effect of Peroxide Modification on Melt Fracture of Linear Low Density Polyethylene during Extrusion. Polym. J..

[44] Vaes D., Van Puyvelde P. (2021). Semi-Crystalline Feedstock for Filament-Based 3D Printing of Polymers. Prog. Polym. Sci..

[45] Liu F., Vyas C., Poologasundarampillai G., Pape I., Hinduja S., Mirihanage W., Bartolo P. (2018). Structural Evolution of PCL during Melt Extrusion 3D Printing. Macromol. Mater. Eng..

